# Selections

**Published:** 1893-03

**Authors:** 


### Selections.

### A NEW COCA BASE AS A LOCAL ANÆSTHETIC.

Dr. A. P. Chadbourne (*Therapeutic Gazette*) says:

A new coca base has recently been isolated by Giesel from the leaves of the small leaved coca plant of Java. Liebermann has proved that this base is benzoyl $\psi$ tropeine, which bears no relation to the cocaine group, but is chemically closely related to atropine.

In its local action the new alkaloid is a connecting link between the true local anæsthetics (cocaine) and the "anæsthetica dolorosa" of Liebreich.

In the experiments now described, the hydrochlorate of benzoyl $\psi$ tropeine was used, the alkaloid itself being insoluble in water. For brevity the name "tropsin" is here given to the drug.

*Experiments on frogs* show the following chief differences between tropsin and cocaine:

1. Tropsin is less than half as toxic as cocaine.
2. It also produces local anæsthesia more rapidly.
3. Individual susceptibility to the drug varies but little, and so unexpected poisoning from a small dose seldom, if ever, occurs.
4. Recovery is quicker from tropsin than from cocoaine.
5. Symptoms of irritation do not follow its use.

*Experiments on rabbits* show:

1. But slight individual susceptibility to its toxic action. There is, however, some individual difference in the nerve-centres most affected in different cases.
2. Tropsin is much less than half as toxic to rabbits as cocaine.

3. Cardiac depressant action is also less marked, and even after stand-still has been produced the heart may be recovered by electrical stimulation.

4. Complete anæsthesia is more quickly produced by tropsin, but is of shorter duration.

5. After instillation into the eye, a slight hyperæmia may be produced for a few moments, but no other signs of irritation, and no ischæmia.

6. Mydriasis is inconstant and slight.

7. A toxic dose of tropsin produces, like cocaine, a marked rise of temperature.

8. Daily repetition of the dose causes marked diuresis, but urine is normal save for low specific gravity and pale color.

*Practical Tests on the Human Subject.*—Professor Schweigger, of Berlin, after several months' experience with tropsin in eye surgery, reports that—

1. A three-per-cent. solution produces complete corneal anæsthesia more rapidly than cocaine. Iridectomy could be done painlessly two minutes after putting three drops into the eye.

2. Anæsthesia lasts from three to six minutes foi each instillation, and no further prolongation can be produced save by a fresh dose.

3. Mydriasis is absent, or but slight.

4. Ischæmia never occurs, but sometimes there is a passing slight hyperæmia, and a little smarting unless normal saline solution be used as a solvent.

5. No injurious symptoms were ever observed.

6. In removal of foreign bodies, tropsin seems, from its quicker action, far preferable to cocaine.

Dr. Silex, assistant in the Polyclinic, has obtained similar results, and has painlessly performed tenotomy within half a minute from applying a three-per-cent. solution of tropsin.—*American Lancet.*

---

### DR. FRIEDRICHS ON NITRATE OF SILVER.

[In the proceedings of the American Dental Association of 1879, Dr. Friedrichs, of New Orleans, read a paper on "Erosion of the Teeth," from which we make the following extract.—Ed.]

After quoting from Dr. Garretson to prove the teeth have a vital action and recuperative power, he says, "Now, from the above premises it is self-evident, if we can bring assistance to the inherent vital force of a tooth, this disease—*erosion of the enamel*—can be cured. The agent to accomplish it is nitrate of silver, for when applied to an eroded part of a tooth it puts forth its fiat and says, 'So far shalt thou go and no farther.' The salt, when brought in contact with the organic matter of the teeth, is decomposed; the oxide of silver is deposited,—an insoluble and inert substance,—which protects and relieves these portions of the teeth of their hypersensitiveness, antagonizes the action of the morbific influences, and assists the 'vis medicatrix naturæ' to eradicate the disease. The method of using it is to chip from a solid stick of nitrate of silver a piece about the size of a pin's head, applying this to the eroded part of the tooth, moving it over the surface until dissolved or decomposed, taking care to keep it from coming in contact with the gums as much as possible. If after a week's time the sensitiveness to the touch has not been allayed, the application must be repeated at intervals until this is attained. No danger need be apprehended of its too frequent use, as no injury to the teeth can possibly ensue, for the escharotic operation of lunar caustic is always superficial, and it is almost impossible to make it act to a great depth. The salt produces, along with its excitant effect, contraction of the tissues; or, in other words, acts as an astringent, and this property also constitutes one of its therapeutic recommendations."

---

### OXYCHINASEPTOL, OR DIAPHTERIN.

LEMBACH and Schleicher (*Correspondenz-Blatt für Schweizer Aerzte*, November 1, 1892) report the discovery of a new antiseptic. As a germicide it is better than phenol and lysol in the form of a two- or three-per-cent. solution. Instruments that are not nickelled are blackened by its use. It has the advantage over carbolic acid from the fact that it is easily transported, either in the form of powder or tablets, and its peculiar yellow color prevents it being mistaken for any other preparation. It is chemically clean and its action easily controlled. The preparation is not poisonous. A watery solution is perfectly clear, and there is no evaporation. Kronacher recommends its use in surgery in strengths of one-half- to two-per-cent. solutions. It is a most excellent dressing in cases of burns. There is never any irritation about the edges of wounds after its use, but occasionally patients complain of a slight burning sensation. The solutions do not affect the hands of the operator as do sublimate and carbolic solutions. Its greatest application is to be found in the treatment of nasal and aural troubles.—*University Medical Magazine.*

### ASAPROL.

Stackler (*Bulletin Génèrale de Thèrapie*, 1892) has a note on his new antiseptic. Asaprol is a calcium salt of naphthol and monosulphonic acid, and occurs in the form of a white powder, very soluble in water and alcohol. The tests for its presence in the urine are the same as those for naphthol. Asaprol is a toxic to rabbits in the proportion of fifty centigrammes to each kilo of body weight. Smaller doses are well borne. It has been tested clinically in two cases of influenza, with high temperature and great pain, with the greatest success. In one case all symptoms were relieved in thirty-six hours, and in the other in forty-eight hours, although quinine and antipyrine had been given without avail. The drug proved equally efficient in the various forms of rheumatism, especially in the acute articular variety.

The drug has also been used with good results in gout, asthma, furunculosis, anthrax, various infective conditions, tonsillitis, etc. Gradually increasing doses are recommended. Thus, for adults the dose will be two grammes on the first day, three on the second, and four on succeeding days, to be again gradually reduced when the anti-thermic action has been obtained. The drug should be given with plenty of water, so as to encourage diuresis. It may also be given by the rectum in doses of from two to seven grammes. The drug is incompatible with alkaline iodides, sulphates, and with most of the alkaline salts.—*University Medical Magazine.*

---

### AGATHIN—A NEW ANTI-NEURALGIC.

Ross (*Deutsche Medicinische Zeitung*, No. 50, 1892) reports his results of the use of agathin in relieving pain. In produces no evil effects by long-continued use. It is tasteless, insoluble in water, but soluble in alcohol and ether, and melts at a temperature of 74° Celsus. The best results are obtained by the use of one-half gramme three times daily. In the pains of neuralgia and rheumatism it is especially valuable. Often from four to six grammes are required before the full effects are noticed.—*University Medical Magazine.*

